# Clinical Manifestations and Pathogenesis of Acute Necrotizing Encephalopathy: The Interface Between Systemic Infection and Neurologic Injury

**DOI:** 10.3389/fneur.2021.628811

**Published:** 2022-01-04

**Authors:** Priya Shukla, Abby Mandalla, Matthew J. Elrick, Arun Venkatesan

**Affiliations:** Department of Neurology, Johns Hopkins University School of Medicine, Baltimore, MD, United States

**Keywords:** encephalitis, COVID-19, influenza, ANE, RanBP2, nucleocytoplasmic transport

## Abstract

Acute necrotizing encephalopathy (ANE) is a devastating neurologic condition that can arise following a variety of systemic infections, including influenza and SARS-CoV-2. Affected individuals typically present with rapid changes in consciousness, focal neurological deficits, and seizures. Neuroimaging reveals symmetric, bilateral deep gray matter lesions, often involving the thalami, with evidence of necrosis and/or hemorrhage. The clinical and radiologic picture must be distinguished from direct infection of the central nervous system by some viruses, and from metabolic and mitochondrial disorders. Outcomes following ANE are poor overall and worse in those with brainstem involvement. Specific management is often directed toward modulating immune responses given the potential role of systemic inflammation and cytokine storm in potentiating neurologic injury in ANE, though benefits of such approaches remain unclear. The finding that many patients have mutations in the nucleoporin gene *RANBP2*, which encodes a multifunctional protein that plays a key role in nucleocytoplasmic transport, may allow for the development of disease models that provide insights into pathogenic mechanisms and novel therapeutic approaches.

## Introduction

In the setting of acute infection, central nervous system (CNS) dysfunction may ensue through a variety of mechanisms. Direct infection of the CNS can result in acute encephalitis, and is characterized by changes in mental status, focal neurological findings, and, importantly, evidence of inflammation by neuroimaging or spinal fluid analysis (1). On the other hand, there is growing recognition of infectious conditions that result in encephalopathy without evidence of overt CNS inflammation (2). In such conditions, which include sepsis-associated encephalopathy, influenza-associated encephalopathy and febrile infection-related epilepsy syndrome, the pathogenic processes that lead to acute brain dysfunction remain poorly defined. A particularly dramatic example of an infection associated encephalopathy is acute necrotizing encephalopathy (ANE), in which necrosis of the deep gray matter of the brain occurs following systemic infections such as influenza or SARS-CoV-2 (3, 4). Importantly, the identification of mutations within affected families- most commonly in the protein *RANBP2* (5)- may shed light on the neuropathogenesis of this devastating condition.

## Clinical Presentation and Outcomes

Acute necrotizing encephalopathy (ANE) is characterized by rapid neurological deterioration following a febrile systemic illness. While most commonly reported in young children, it can also affect adults. Although the most commonly found infectious trigger is influenza, other pathogens, including SARS-Cov-2 and human herpes viruses, have been associated with ANE (Table 1). It is intriguing that such a wide array of pathogens has been linked to ANE despite the enormous variability in their virulence–some, such as rhinovirus typically cause only mild disease in humans, while others, such as dengue virus, are associated with severe, and often fatal, systemic disease. From the initial descriptions by Mizuguchi and colleagues (6–8), a key characteristic is the acute development of multifocal bilateral and symmetrical necrotic lesions, most commonly involving the deep gray matter. Seizures are commonly reported (up to 50% of cases), and intracerebral hemorrhage, cerebral edema, and coma can develop (5, 7, 9). Fever is present in about 2/3 of cases, and systemic manifestations, including respiratory failure, liver dysfunction, and diarrhea can also occur. A systemic inflammatory response syndrome leading to multiple organ failure has been reported (10–12).

**Table 1:** Infections associated with ANE.

**Infectious trigger**	**Number of cases**	**Cited cases**
Unknown Pathogen	26	Zhou (2014), Kobayashi (2019), Narra (2015), Wetzburger (1998), Sharma (2019), Hassanzadeh (2017), Akiyoshi (2006), Mastroyianni (2006), Bassuk (2003), Porto (1999), Sell (2016), Lee (2017), Marco (2010), Marco (2010), Dangi (2020), Oh (2004), Weng (2010), Wolf (2013), Ravid (2001), Shibata (2019), Akiyoshi (2006), Dai (2016), Ueno (2002), Hayakawa (2007), Soriano-Ramos (2018), Manara (2006)
H1N1	19	Ormitti (2010), Anand (2015), Offiah (2012), Aruajo (2016), Martin (2010), Lyon (2009), Komur (2011), Mariotti (2010), Isikay (2016), Mungaomklang (2016), Koh (2019), Koh (2019), Ochi (2018), Demir (2019), Demir (2019), Demir (2019), Abdelrahman (2019), Howard (2018), Howard (2018)
Influenza A	18	Kumakura (2011), Offiah (2012), Gika (2010), Lee (2012), Kirton (2005), Ichiyama (2003), Vourdris (2001), Okumura (2006), Marco (2010), Vargas (2012), Munakata (2000), Fasano (2008), Lee (2011), Watanabe (1998), Shinohara (2011)
HHV-6	13	Yoshida (2013), Kubo (2006), Huang (2020), Skelton (2008), Sell (2016), Shinohara (2011)
Influenza B	9	Bloch (2015), Bloch (2015), Onozawa (2018), Huang (2004), Sazgar (2003), Nishimura (2016), Koh (2019), Taniguchi (2017), Samanta (2019)
SARS-CoV-2	5	Al Mazrouei (2020), Elkady (2020), Delamarre (2020), Dixon (2020), Poyiadji (2020)
HHV-6B	3	Kansagra (2011), Kawamura (2013), Ohsaka (2006)
VZV	3	Kirton (2005), Tran (2001), Tran (2001)
Enterovirus	2	Tabarki (2013), Orgun (2020)
Mycoplasma Pneumoniae	2	Lee (2017), Shinohara (2011)
Rotavirus	2	Kirton (2005), Shinohara (2011)
Gastrointestinal Infection	2	Saitoh (2012), Salehiomran (2013)
Plasmodium Vivax	1	Yadav (2009)
Streptococcus Pneumoniae Bacteremia	1	Huber (2020)
Parainfluenza	1	Mastroyianni (2003)
Victoria Lineage Influenza B	1	Larsh (2020)
Rhinovirus	1	Alawadhi (2018)
Viral Bronchitis	1	Nishimura (2016)
Diptheria, Tetanus, Pertussis Vaccine	1	Aydin (2010)
Coxsackie Virus	1	Fasano (2008)
EBV	1	Lin (2019)
Respiratory Syncytial Virus (RSV)	1	Shinohara (2011)
Adenovirus	1	Shinohara (2011)
Dengue Fever	1	Abbas (2017)

Radiographic findings typically reflect edema, necrosis, and in some cases, hemorrhage in the deep gray matter (13). While computed tomography (CT) on presentation may be normal very early on (14), findings typically evolve rapidly such that subsequent scanning demonstrates deep gray hypodensities. Brain magnetic resonance imaging (MRI) often demonstrates restricted diffusion in affected areas, along with hypointensities on T1 weighted imaging and hyperintensities on T2 weighted imaging. Susceptibility weighted imaging (SWI) sequences on MRI may demonstrate evidence of microhemorrhages even if frank hemorrhage is not observed on CT (13). Notably, ANE must be distinguished from a number of other conditions that can result in acute neurologic manifestations and deep gray matter abnormalities on imaging. Japanese encephalitis virus and other neurotropic arboviruses can directly invade the CNS and account for a similar clinical picture, as can toxic disorders such as carbon monoxide poisoning, mitochondrial disorders such as Leigh disease, and vascular conditions such as deep venous sinus thrombosis (15–17). Indeed, the differential diagnosis in a patient with suspected ANE is quite broad.

Routine laboratory examination often demonstrates evidence of liver dysfunction, with elevated levels of aspartate aminotransferase, alanine aminotransferase and lactate dehydrogenase; in contrast, hyperammonemia is rarely reported and its absence may help to distinguish ANE from some mimics (18). Thrombocytopenia is also commonly seen, and may be associated with disseminated intravascular coagulation. CSF protein is typically elevated, often > 100 mg/dL, likely as a consequence of neuronal damage and necrosis (7, 8). Pleocytosis of white blood cells is exceedingly rare, arguing against ANE as a primary inflammatory disorder of the CNS. Furthermore, there is seldom evidence of viral infiltration of the CNS (19). These findings suggest an infection-triggered brain injury that is neither due to CNS infection nor substantial inflammation.

**Figure 1:**
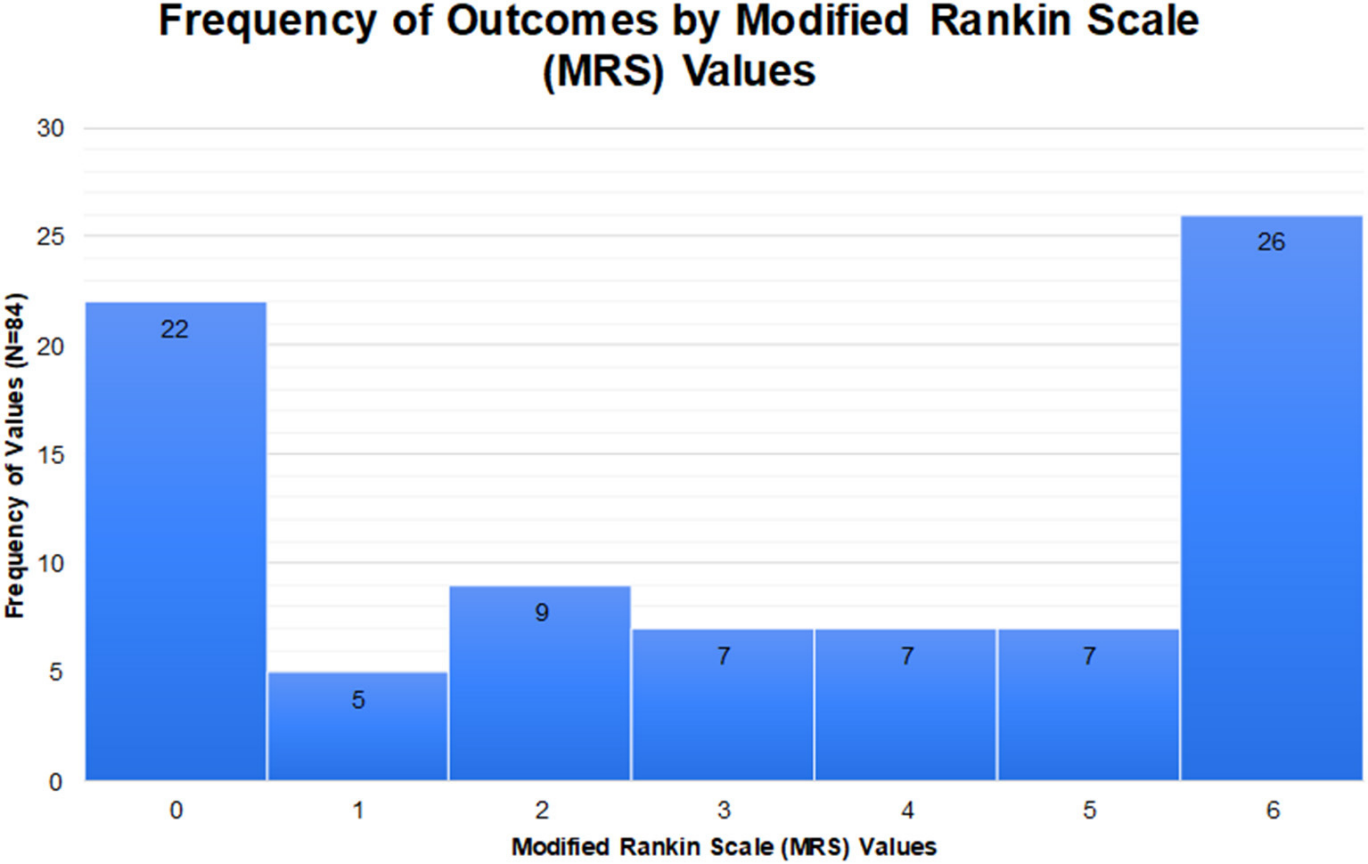
Outcomes of ANE from 84 cases in available literature where modified Rankin scores could be ascertained.

ANE outcomes span the spectrum from complete recovery to death, though the majority of outcomes are severe (Figure 1). Even in cases of complete recovery, prolonged hospitalization and rehabilitation may be required. Although it remains unclear exactly what contributes to severe outcomes, involvement of the brainstem is associated with an increased risk of death (18, 20). In an effort to better characterize severity and outcome in ANE, a severity score, termed ANE-SS, has been developed (21). The ANE-SS incorporates the presence of shock (+3 points), brainstem lesions (+2 points), older age (+2 points if above 4 years of age), thrombocytopenia (+1 point), and elevated CSF protein (+1 point). Notably, in a study of 73 ANE patients, ANE-SS correlated with outcomes in patients assessed at a year or longer following the acute presentation (21).

## ANE vs. ANE1

In 2009, Neilson and colleagues identified a genetic predisposition for ANE in the nucleoporin gene *RANBP2* (22). This finding prompted the recategorization of ANE patients with missense mutations in the leucine rich region of RanBP2 as ANE1. While a threonine to methionine mutation (T585M) accounts for the majority of familial or recurrent ANE cases, other mutations in the RanBP2 gene have been reported in the setting of ANE, including T653I, I656V, T681C, and Lys1665Glu (22–25). A different genetic abnormality- a chromosomal translocation resulting in fusion of part of the RANBP2 gene with the anaplastic lymphoma kinase gene- has been associated with inflammatory myofibroblastic tumor, but not with ANE (26).

The T585M mutation is inherited in an autosomal dominant fashion with incomplete penetrance (22). Mutations have also arisen de novo in children whose parents are unaffected (19). Interestingly, genotyping of families affected by ANE1 often shows unaffected carriers, highlighting the complexity of disease pathogenesis (27). Furthermore, *RANBP2* mutations in a consanguineous family without previous history of ANE raises the possibility of recessive inheritance of some mutations (22). Of note, not all recurrent or familial ANE cases are associated with mutations in RanBP2 (28–30). Mutations in other genes such as SCNIA R1575C and carnitine palmitoyltransferase II have also been associated with ANE (31, 32). There also appears to be a correlation between HLA genotypes and ANE susceptibility (33, 34).

Although similar in many respects, there are some distinctions between ANE and ANE1 (Table 2). ANE1 patients often have lesions in regions such as the amygdalae, hippocampi, and medial temporal lobes, that are not typically seen in other ANE patients (22). While ANE patients commonly exhibit elevated serum transaminases this is less often the case in ANE1 patients (8, 22). One prominent distinction between ANE1 and ANE is the rate of recurrence, as sporadic ANE patients seldom have recurrent episodes.

**Table 2:** Clinical characteristics of ANE vs. ANE1.

**Sporadic ANE**	**Both**	**ANE1**
- Cerebral periventricular white matter and cerebellum lesions - Frequent elevation of transaminases - Systemic organ damage	- Elevated CSF protein - Symmetric bilateral thalamic lesions	- High rate of recurrence - Potentially lower rate of seizures - Lesions in external capsule, claustrum, medial temporal lobes, amygdalae, hippocampi, medial temporal lobes

## RanBP2

**Figure 2:**
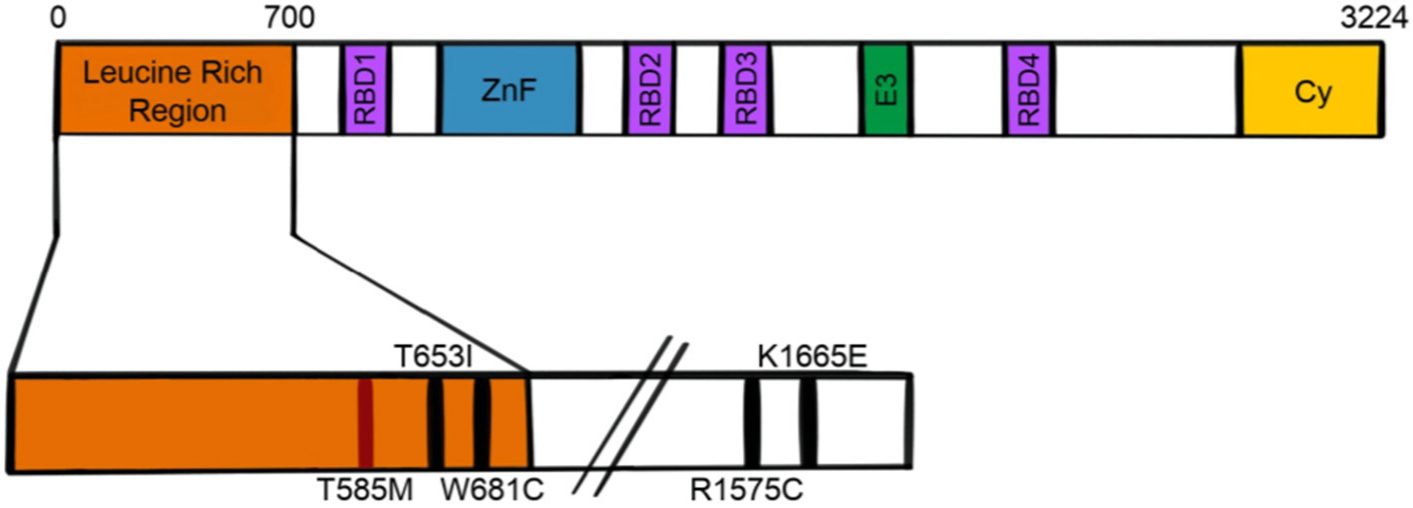
Schematic of RanBP2 protein. RBD, Ran binding domain; ZnF, zinc finger; E3, E3 SUMO ligase domain; Cy, cyclophilin homologous domain.

RanBP2 is a 358kDa, multi-domain, cytoplasmic nucleoporin that influences a multitude of cellular functions (35–37). Like several other nucleoporins, RanBP2 contains FG/FxFG repeats which facilitate nucleocytoplasmic transport (35, 37, 38). It also contains an N-terminal leucine rich region or leucine domain, a zinc finger domain containing eight zinc finger motifs, four Ran binding domains, a kinesin binding domain, an E3 SUMO ligase domain, and a cyclophilin homologous domain (Figure 2). While several reports indicated localization at the axon initial segment (AIS) of neurons (39, 40), the use of more specific antibodies has recently demonstrated that endogenous RanBP2 is not found in the AIS (41).

### RanBP2 and Nucleocytoplasmic Transport

**Figure 3:**
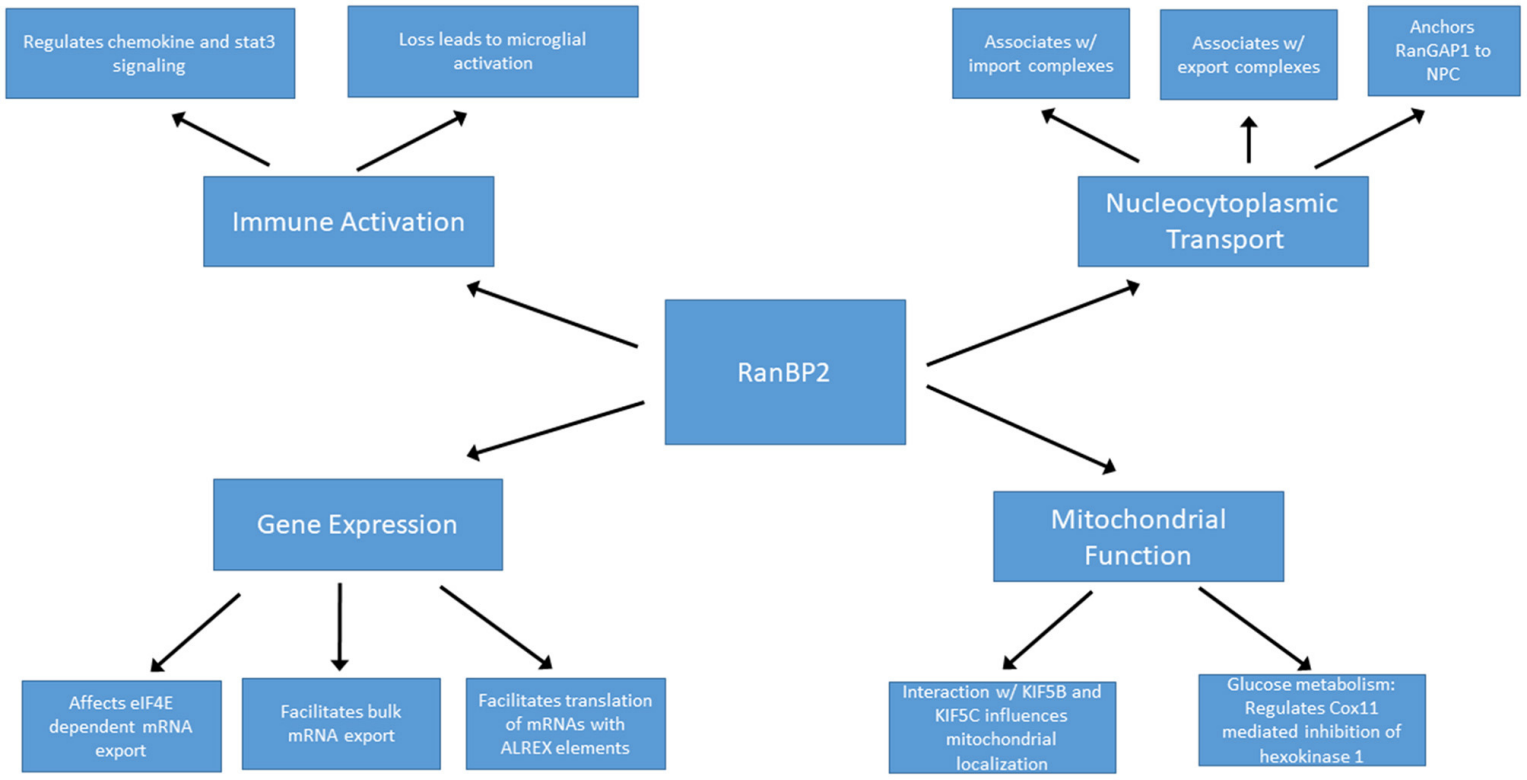
RanBP2 is a multifunctional protein.

RanBP2 is localized to the cytoplasmic filaments of the nuclear pore complex, and plays a major role in nucleocytoplasmic transport (Figure 3). The directionality of nucleocytoplasmic transport relies upon maintenance of the Ran gradient. Ran is a Ras-related GTP hydrolase. The Ran gradient is established by the Ran guanine activating protein RanGAP1 by stimulating the hydrolysis of RanGTP to RanGDP in the cytoplasm, and by the guanine nucleotide exchange factor RCC1 exchanging RanGDP for Ran GTP in the nucleus (42, 43). RanBP2 anchors RanGAP1 at the nuclear pore complex, thus helping to maintain the critical Ran gradient (44–47). RanGAP1 is first conjugated to SUMO1 by the E2 SUMO-conjugating enzyme Ubc9. In turn, sumoylated RanGAP1 and Ubc9 interact with the internal repeat domains of RanBP2 to form a stable RanBP2/RanGAP1-SUMO1/Ubc9 complex that remains associated with the cytoplasmic filaments of the NPC and also functions as an E3 SUMO ligase (48).

RanBP2 plays roles in nucleocytoplasmic transport of protein and RNA cargoes. The nuclear export receptor CRM1 interacts with the zinc finger and FG-repeat domains of RanBP2 during protein export. The release of exported cargo is facilitated by the interaction of Ran-GTP with the Ran binding domains of RanBP2. This destabilizes the CRM1-RanGTP interaction leading to the release of exported cargo from CRM1 on the cytoplasmic side of the NPC (49, 50). Depletion of RanBP2 impairs nuclear export, though the other cytoplasmic filament nucleoporins Nup214 and Nup88 are also involved in this process and can partially compensate for loss of RanBP2 (51). mRNA export requires the interaction of the RanBP2 FG repeat domain with the NFX1-p15 heterodimer that functions as the export adapter for mRNAs (52). The role of RanBP2 in protein import has been controversial. RanBP2 deletion from mouse embryonic fibroblasts leads to decreased protein import associated with reduced docking of importin-β to the NPC (53). Depletion of RanBP2 in human cell lines resulted in the shift of nucleocytoplasmic transport (NCT) reporter constructs and a subset of endogenous nuclear proteins to the cytoplasm (42, 54, 55). By contrast, deletion of RanBP2 in *Xenopus* oocytes had no significant effect on protein import (56). Relative to its essential role in protein and RNA export, RanBP2 likely plays a relatively minor and species-specific role in facilitating nuclear import of protein cargoes.

Mice lacking RanBP2 exhibit embryonic lethality and conditional knockout of RanBP2 in cultured mouse embryonic fibroblasts resulted in progressive cell death in association with disrupted NCT. Of note, the presence of the N-terminal domain of RanBP2, which contains the leucine rich region where ANE mutations typically occur, is sufficient for viability of these cells (53). This domain, along with the FG repeats which associate with NFX1 mRNA shuttling protein, are both critical for efficient mRNA export (52, 53).

### RanBP2 and Regulation of Gene Expression

In addition to the aforementioned roles in bulk mRNA transport, RanBP2 may have additional functions in the regulation of specific subsets of mRNAs. Through its zinc-finger domain, RanBP2 facilitates translation of mRNAs containing alternative mRNA nuclear export (ALREX) elements, including ER proteins, secretory proteins and likely even mitochondrial proteins (57). Depletion of RanBP2 in U2OS cells resulted in a dramatic and selective reduction of the production of secretory proteins (57). This may be of particular importance in the setting of ANE, since elevated secreted cytokines may play a central role in disease pathogenesis (discussed below).

In addition, RanBP2 may indirectly regulate nuclear export of specific mRNAs. For example, the eukaryotic translation initiation factor eIF4E promotes export of a subset of mRNAs from nucleus to cytoplasm. Overexpression of RanBP2 limits this export pathway, likely by slowing the release or recycling of export factors Depletion of RanBP2, on the other hand, results in increased export of eIF4E target mRNAs (58). RanBP2 also interacts with argonaute (AGO) proteins and helps target certain mRNAs, including the proinflammatory cytokine interleukin-6, for silencing through miRNA induced silencing complex (RISC) (59). These unique and opposing effects exhibit the importance of RanBP2 in maintaining and regulating gene expression profiles at the mRNA export and translational levels. Moreover, alteration of export or silencing of specific mRNAs in the setting of RanBP2 mutations could play a role in the pathogenesis of ANE. These observations highlight the need to determine whether disease-causing RanBP2 mutations affect export or translation of specific mRNAs.

### RanBP2 and Mitochondrial Function

In photosensory neurons, haploinsufficiency of RanBP2 resulted in mislocalization of mitochondrial hexokinase I and a 50–60% reduction of hexokinase I specifically within the CNS with corresponding deficits in glucose metabolism and ATP production (60), suggesting that regulation of glycolysis by RanBP2 is especially critical for neurons. The kinesin binding domain of RanBP2 interacts with KIF5B and KIFC and influences localization and function of mitochondria, thus potentially further directing cellular metabolism (61). Notably, ANE patients exhibit evidence of mitochondrial dysfunction such as loss of coupling of oxidative phosphorylation (27).

Mitochondria play an important role in innate immune signaling. For example, toll-like receptors, nuclear oligomerization domain like receptors, and RIG-I like helicase receptors rely upon mitochondrial signaling pathways (62, 63). When activated, the NLRP3 inflammasome uses the mitochondria as a scaffold where it responds to infection or cellular damage by regulating proinflammatory cytokine secretion including IL-1ß and IL-18 (62, 64). Thus, RanBP2 mutations in ANE that affect mitochondrial function may not only result in alterations in metabolism and energy production but also immune signaling.

### RanBP2 and Immune Activation

Ablation of RanBP2 can lead to marked derangements in innate immune signaling through mechanisms that are not yet well defined. Broadly speaking, RanBP2 loss in retinal ganglion neurons causes activation of microglia (65). From a mechanistic standpoint, loss of RanBP2 leads to intracellular sequestration of the matrix metalloproteinase Mmp28 such that Mmp28 is no longer able to suppress production of the chemokine Ccl6. Ccl6, in turn, acts as a macrophage chemoattractant (66) and the lack of Mmp28 has been associated with increased macrophage chemotaxis and chemokine production (67, 68). In motor neurons, on the other hand, loss of RanBP2 leads to a posttranscriptional decrease in Mmp28 which is associated with dampened microglial and macrophage inflammatory responses (69). Moreover, marked derangements were found in transcription, translation, and intracellular localization of a number of molecules involved in the innate immune response, including Cxcl14, Cxcl12, and Stat-3. In cone photoreceptors, ablation of RanBP2 resulted in early and marked upregulation of Mmp11, which can cause damage to the secreting cell itself in addition to neighboring cells, potentially leading to a feed-forward process of neuronal injury (70). Overall, the loss of RanBP2 is associated with derangement of innate immune mechanisms in a complex and cell type specific fashion that may, in part, be accounted for by alterations in local matrix metalloproteinase expression and function.

## Cytokine Storm, the Blood Brain Barrier, and ANE

Dysregulation of cytokine production appears to be a common feature of ANE. In the setting of infection, the innate immune system acts as a “first responder,” elaborating an array of pro-inflammatory mediators including interferons, interleukins, and chemokines in an effort to clear the pathogen (71, 72). These immune cascades are, under normal circumstances, tightly controlled with respect to amplitude and duration due to feedback mechanisms that fine-tune the response (73, 74). Unrestrained activation, however, can result in marked systemic inflammation with deleterious consequences to the host, a condition termed “cytokine storm.” The finding of elevated cytokine levels, sometimes markedly so, in ANE has raised the possibility that cytokine storm may play a central role in disease pathogenesis (75).

While elevated cytokine levels in the serum have been reported in the setting of ANE (76–79), there is less data on CSF cytokine levels (77, 78, 80). However, it is likely that high serum cytokine levels would contribute to high CSF cytokine levels in the setting of breakdown of the blood brain barrier (BBB). Indeed, cytokines such as TNFalpha and IL-6, which are elevated in ANE, have been shown to cause breakdown of the BBB both *in vitro* and *in vivo* (81–86). *In vitro*, administration of IL-6, TNFalpha and IL-1B to rat cerebral endothelial cells reduced trans-endothelial electrical resistance by 50%, reflecting perturbations in tight junction stability. These cytokines cause cerebral endothelial cells to produce eicosanoids, such as thromboxane A_2_ and prostaglandin E_2_, which interact with thromboxane A2 receptors (83, 87). This interaction causes vasodilation and increased permeability of the BBB (88). Notably, the effects of IL-6, TNFalpha, and IL-1B on permeability could be prevented by a cyclooxygenase inhibitor, and could be reversed over time (83). TNFalpha is also known to cause ultrastructural changes to tight junctions (89), likely enhancing their permeability. A report that gadolinium enhancement precedes some of the other neuroradiological manifestations of ANE (90) lends further support to the hypothesis that a surge of cytokines in the periphery may alter the BBB and directly contribute to the pathogenesis. Intra-CNS production of pro-inflammatory cytokines may also occur in some cases; indeed, in one report levels of IL-6 that were over 100 fold greater in the CNS than serum during the acute phase of ANE and 8 fold higher during the late phase (77).

Elevated cytokines are a common feature in several other types of encephalopathy/encephalitis, including influenza associated encephalitis (IAE). Notably, serum levels of IL-6 were predictive of influenza associated encephalitis disease severity (91). It is believed that elevated IL-6 precedes neurological symptoms, and thus may not necessarily be elevated during or after encephalopathy. Patients with lowest maximal levels of IL-6 had the best outcomes while patients with IL-6 levels over 15,000 pg/mL did not survive, despite high dose corticosteroid treatment. Brainstem dysfunction was associated with IL-6 levels 6,000 pg/mL and over, while cases without brainstem involvement peaked around 150 pg/mL. Thus, it is possible that IL-6 may play a central role in a range of infection-associated encephalopathies.

Cytokines are known to affect neuronal function and high levels of CSF proinflammatory cytokines could directly contribute to the neurological damage seen in ANE (81, 84). For example, elevated proinflammatory cytokine levels can increase excitatory glutamatergic neurotransmission while simultaneously reducing inhibitory GABAergic neurotransmission, increasing the risk of excitotoxicity (92). Interestingly, cytokines such as TNFalpha and IL-6 are also released after limbic seizures in rats (93), thus potentially fueling the existing positive feedback loop of cytokine production. IL-6 is also produced after cellular injury (94), thus potentially contributing to a deleterious positive feedback loop.

## Management

While a number of treatments have been suggested for ANE, there is limited evidence to support any individual approach.

### Corticosteroids

Given the potential role of proinflammatory cytokines in driving the disease process, some form of immunomodulatory therapy such as corticosteroids or intravenous immunoglobulin is often used. It is possible that the timing of immunomodulatory therapy may be critical. In seven of twelve patients who received steroid treatment within 24 h of symptom onset, good outcomes were noted in comparison to poor outcomes in all five patients without early steroid intervention (20). Unfortunately, among those with brainstem lesions, outcomes were poor and no treatment showed any correlation with outcome. In another case, an 8-year-old girl with influenza A presented with fever and generalized tonic-clonic seizures, raising the possibility of impending ANE (32). Her initial CT and MRIs appeared normal and she was treated with pulsed methylprednisolone (20 mg/kg/day for 3 days) and high dose gamma-globulin therapy (1 g/kg/day for 2 days) 4 h after her seizure. Twelve hours after her seizure, imaging revealed bilateral thalamic and brainstem lesions consistent with ANE. Notably, this patient made a full recovery, despite the presence of brainstem lesions. This case also demonstrates that corticosteroid therapy before the onset of brainstem lesions might alter the course of disease progression and improve outcome. Similarly, in a patient with recurrent ANE1, treatment within 24 h of onset with 20 mg/kg/day methylprednisolone for 5 days and then prednisone 2 mg/kg/day for 6 weeks led to greater improvement with more rapid recovery than in prior episodes in which immunomodulatory therapy was not given (95).

### IL-6 Blockade–Tocilizumab

Since IL-6 appears to be the most widely and highly elevated cytokine, attempts to control IL-6 levels represent a potentially rational approach to ANE management and treatment. IL-6 levels correlate with severity of outcome (96), providing further rationale for targeting IL-6 levels. In one study, patients with high risk ANE (ANE-SS = 5) with brainstem lesions, but without RanBP2 mutations were treated with Tocilizumab, a monoclonal antibody targeting the IL-6 receptor, 18–32 h after the onset of neurological symptoms. Two patients recovered completely, and the third only had mild sequelae likely due to profuse hemorrhage early in the disease course. Furthermore, while previous studies have linked brainstem lesions with extremely poor outcomes patients in this study showed remarkable outcomes with tocilizumab treatment despite the presence of brainstem lesions. Interestingly, positive outcomes were noted in the setting of tocilizumab treatment even if initial IL-6 levels were considered normal; while this may potentially be due to the variable course of IL-6 levels during ANE, the normal IL-6 levels call into question the mechanism of potential benefit.

### Hypothermia

Hypothermia reduces brain metabolism and cerebral blood flow, and has anti-inflammatory effects (97, 98). In the setting of ischemia after experimental stroke, neuroprotection is achieved in part through reduction of neutrophil infiltration and microglial activation (98–100), the latter of which may be particularly relevant in ANE. Furthermore, hypothermia is able to decrease nuclear translocation of the pro-inflammatory transcription factor NFkB and reduce levels of the proinflammatory cytokines most commonly and highly elevated in ANE, including IL-6 and TNFalpha (101, 102). Several small studies to date have combined the use of hypothermia with other anti-inflammatory agents in the management of ANE (103, 104).

### Serine Protease Inhibitors

Serine proteases play a critical role in the inflammatory response (105) and thus blocking their activity can attenuate hyperinflammatory responses. Urinary Trypsin Inhibitor, or Ulinastatin, is a serine protease inhibitor that has been used in Japan to treat acute sepsis, and disseminated intravascular coagulation (DIC) (106–108), the latter of which is a feature commonly seen in ANE patients. In animal models of sepsis, treatment with serine protease inhibitors (bikulin and ulinastatin) reduces TNFalpha, IL-6 and a multitude of other inflammatory mediators (106, 109). Furthermore, ulinastatin inhibits phosphorylation of p38 MAPK, resulting in reduced expression of pro-inflammatory genes such as TNFalpha (110). Other studies show that ulinastatin suppresses JNK/c-Jun signaling (111). Ulinastatin has also been shown to improve experimental autoimmune encephalomyelitis by reducing oligodendrocyte apoptosis and demyelination and by reducing levels of cytokines such as IL-1B and IL-6 (112).

Ulinastatin has also been evaluated in humans. In patients with sepsis, ulinastatin has also been shown to reduce serum levels of TNF-alpha and IL-6 and other pro-inflammatory mediators while increasing levels of the anti-inflammatory IL-10 (106, 107, 113). Notably, endogenous protease inhibitors are usually synthesized in the liver (114), and as previously mentioned liver dysfunction is also commonly noted in ANE. Thus, it is plausible that liver dysfunction in ANE reduces the output of serine protease inhibitors, further exacerbating the already heightened immune response.

### Insights From Related Encephalopathies

Nup214, which like RanBP2 is a nucleoporin localized to the cytoplasmic filaments of the NPC, is also associated with an acute or progressive encephalopathy. Two independent groups have reported a total of three families with homozygous or compound heterozygous mutation of *NUP214*. The affected children have hypotonia, global developmental delay, and either cerebellar hypoplasia or microcephaly. Patients in two of the families experienced dramatic developmental regression following routine viral respiratory infections, characterized by seizures, hyperkinetic movement disorder, and progressive volume loss in the cerebral cortex and cerebellum, and lesions in the thalami. The third family showed an early onset neurodegenerative phenotype without apparent distinct episodes of regression or provoking factors. The findings were accompanied by altered nuclear protein import, RNA export, and decreased cell survival in patient skin fibroblasts. No effective therapies are yet known for *NUP214-*associated encephalopathy (115, 116).

As previously described, several other acute encephalopathies can also affect the deep gray matter of the brain. Mitochondrial disorders, in particular, share some common clinical features with ANE and it is possible that the failure of neuronal energetics seen in these conditions may also contribute to the pathophysiology ANE. While treatment for mitochondrial disorders consists mainly of supportive care, there has been growing interest in specific therapies focused on correcting or bypassing specific biochemical abnormalities in the setting of known mutations in mitochondrial proteins (117). In addition, broad spectrum approaches including supplementation with agents such as creatine and coenzyme Q have been evaluated, though have not demonstrated proven benefit (118). Notably, following a randomized controlled trial and monitoring of data from a subsequent expanded access program, the antioxidant idebenone was approved to treat visual dysfunction in the setting of the mitochondrial disorder Leber's hereditary optic neuropathy (119, 120). Whether such approaches will benefit patients with ANE is unknown.

## Conclusions and Future Directions

While there is little evidence in ANE for direct viral invasion of the CNS or overt CNS inflammation, much remains unknown regarding the pathogenesis of disease. Growing evidence for systemic derangements in proinflammatory cytokines suggests a potential role for systemic cytokine storm. Many of the currently utilized approaches for management of ANE focus on the hypothesis that dysregulation of systemic cytokines drives disease, though their utility is yet to be proven. There is a need for controlled trials of therapeutics, which is likely to be quite challenging due to the rarity of the condition. The discovery of RANBP2 mutations in familial and recurrent ANE provides the opportunity to identify at-risk cohorts of patients for clinical trials, and may also assist in the development of relevant disease models to identify the functions of RanBP2 central to disease pathogenesis. Given the role of RanBP2 in NCT, dysregulated transport of cargoes between nucleus and cytoplasm may play an important role. However, other cellular processes, such as mitochondrial functioning, may also be directly impacted by RanBP2 and may be particularly important to elucidate given similarities with some mitochondrial disorders in which neurologic injury is triggered by infection. Moreover, given the clinical similarities between ANE and some metabolic and mitochondrial disorders, it will be important to investigate whether the function of RanBP2 is altered in those diseases. Overall, a better understanding of disease pathogenesis in ANE may allow for the development of novel, targeted therapeutics that can limit neurological injury.

## Author Contributions

PS and AV: conceived of the manuscript, drafted, and provided critical revisions. AM: drafted the manuscript. ME: provided critical revisions. All authors contributed to the article and approved the submitted version.

## Funding

This work was funded by NIH R21NS121462 awarded to AV and ME.

## Conflict of Interest

The authors declare that the research was conducted in the absence of any commercial or financial relationships that could be construed as a potential conflict of interest.

## Publisher's Note

All claims expressed in this article are solely those of the authors and do not necessarily represent those of their affiliated organizations, or those of the publisher, the editors and the reviewers. Any product that may be evaluated in this article, or claim that may be made by its manufacturer, is not guaranteed or endorsed by the publisher.
